# Mediating effect of workplace violence on the relationship between empathy and professional identity among nursing students

**DOI:** 10.3389/fpsyg.2022.964952

**Published:** 2022-12-12

**Authors:** Liping Wang, Haiyang Li, Qiongni Chen, Chunhua Fang, Lifang Cao, Li Zhu

**Affiliations:** ^1^Clinical Nursing Teaching and Research Section, The Second Xiangya Hospital of Central South University, Changsha, China; ^2^Department of Kidney Transplantation, The Second Xiangya Hospital of Central South University, Changsha, China; ^3^Department of Cardiology, The Second Xiangya Hospital of Central South University, Changsha, China

**Keywords:** workplace violence, empathy, professional identity, nursing students, mediating effect

## Abstract

**Background:**

Studies have reported that empathy had a positive effect on professional identity (PI) in nursing students. However, little was known about the mechanism underlying this relationship between empathy and professional identity in nursing students.

**Objective:**

The purpose of this study was to analyze in depth the mediating effect of workplace violence (WVP) between empathy and professional identity in nursing students.

**Methods:**

A total of 405 nursing students participated and were investigated using the Chinese version of the Jefferson Scale of Empathy-Health Professional (JSE-HP), the scale of professional identity about nursing students, and the workplace violence Incident Survey in this study. Hierarchical regression was used to analyze the mediating effect of workplace violence on the relationship between empathy and professional identity among nursing students.

**Results:**

The score of nursing students' professional identity was 103.69 ± 17.79. Workplace violence had a significant negative correlation with empathy (*r* = −0.449, *P* < 0.001) and professional identity (*r* = −0.330, *P* < 0.001). Workplace violence accounted for 14.59% of the total mediating effect on the relationship between empathy and professional identity for nursing students.

**Conclusions:**

In this study, the level of professional identity of nursing students was low. Workplace violence had a partially significantly mediating effect on the relationship between empathy and professional identity. Maybe, it was suggested that nursing students' professional identity might be improved and driven by a decrease in workplace violence. Targeted interventions at reducing nursing students' workplace violence should be developed and implemented. In addition, nursing managers and educators should be aware of the importance of empathy and improve professional identity in nursing students.

## Introduction

Shortage of nurses is a perennial problem that is currently being experienced throughout the world (Chen et al., 2020). Nursing students are the successors to nursing careers. Therefore, the nurse scientists should pay additional attention as to whether after their graduation, the nursing students would choose nursing as their career or not (Bakker et al., 2018; McCarthy et al., 2020). On the contrary, an increasing number of nursing students are not continuing in the nursing profession after graduation but are pursuing other careers. Many reasons are attributed to this changeover of careers by nursing students. Some nursing students opine that nurses have a low social status and are not recognized, despite their best humanitarian efforts at bringing succor and relief to patients through their sisterly care. One study shows that the nurses have a low social status and the public image of a nurse is not as good and powerful as a doctor, especially when seen from the perspective of cultural and educational factors (Guo et al., 2017). Yet another impression held about the nursing profession is that the nurses, who do repetitive and routine work to some degree, have to execute the orders from the doctor, which is the adhered-to-practice in many parts of the world (Holroyd et al., 2002). Another section of students after clinical practice have a more intuitive understanding about nursing majors and think nursing work to be an arduous and intense task. The heavy work coupled with physical burden due to working in shifts results in the low professional identity (PI) among Chinese nurses (Feng et al., 2017). Some nursing students have even suffered unexpected maltreatment, such as Workplace violence (WPV), at others' hands during their clinical practice. WPV is usually directed at healthcare teams, but it is now becoming a widespread growing phenomenon targeting nursing students and affecting them as well (Warshawski, 2021). WPV is common in hospitals, with a study showing that about half of the nursing students would have already experienced WPV (Tee et al., 2016). It can be observed that, to some degree, WPV has a deep negative impact on professional identity (PI). Earlier, one study had shown that the lack of PI may be a contributing factor to nursing students leaving the nursing program abruptly, and graduate nurses leaving the nursing profession itself (Deppoliti, 2008). It is equally worrying to note that the overall level of the PI of nursing students is low (Guo et al., 2018; Wu et al., 2020). Empathy is seen as a cognitive ability, which consists mainly in understanding others' thoughts, intentions, and motivations (Di Lorenzo et al., 2019). It is a basic competency of helping relationship and an integral component of person-centered care (Gholamzadeh et al., 2018). Some studies dealing with the empathy of nursing students portray the fact that their empathy is at a low ebb and it needs to be given special attention to reflect the original empathy, as is wont of nurses (Williams et al., 2016; Larti et al., 2018). Consequently, some scholars have even carried out relevant research works on the ability of empathy of nursing students, and found that empathy is a teachable competency (Gholamzadeh et al., 2018). Literature shows that empathy strengthens the relationship between patients and healthcare professionals and also improves the patient's and healthcare professional's satisfaction, which in turn helps to bring out the best clinical outcomes (Petrucci et al., 2016). Therefore, we have reason to assume that empathy affects PI to some extent.

To our knowledge, few studies have explored the role of WPV in mediating empathy and PI in nursing students. Knowledge about the specific role of WPV in the relationship between empathy and PI helps to develop effective interventions to promote nursing students' professional honor and to look for the possibility of a career in nursing. Thus, the purpose of this study was to investigate the mediating effect of WPV on the relationship between empathy and PI.

## Methods

### Study design

This was a cross-sectional study which was conducted at five universities (Central South University, Hunan Normal University, Huaihua College, Xiangnan College, Shaoyang College) in the Hunan province of China from 21 October 2021 to 21 November 2021. Our study adopted the purposive sampling method to gather responses from the nursing students and collect related valuable data. The nursing student participated in the study and completed three different questionnaires. In detail, each student spent about 15–20 min for the questionnaires and signed the informed consent in person.

### Participants and procedures

Inclusion criteria for this study were as follows: Students were included, if they (1) were full-time nursing students, including junior, undergraduate, and postgraduate; (2) were nursing students who had completed an internship, as required by the school of nursing; (3) spoke Chinese and communicated well with others; and (4) had already provided informed consent to participate in this survey. Exclusion criteria for this study were as follows: Students were excluded, if (1) their internship period was <6 months and (2) their absence during the internship period was more than or equal to 1 month.

### Ethical considerations

This study was approved by our hospital's Ethics Committee (No: E202071). We acquired the written consent of all participants to take part in this study. The participants were informed that they could terminate participation in the study, without offering any explanation or think of consequences to their career, at any time during this study. In addition, the questionnaire was conducted anonymously, so the acquired data were kept strictly confidential and used only for the purpose of this study.

### Data collection

The research team that administered the questionnaire to the nursing students comprised five trained researchers. Except for the chairman, two members of this team went as a group to the target university or college. With permission from the headmaster, we, the research team, recruited subjects from department meetings. After obtaining oral consent from these subjects, we then handed out the questionnaires to the nursing students face to face (which also required the participants to give their written informed consent). At this stage, nursing students were explained the purpose and methods of the present study so as to make them ready to fill the questionnaire. Then, the completed questionnaires were retrieved. Ultimately, a total of 410 surveys were distributed.

### Measures

The questionnaire materials included a demographic questionnaire, the WPV Incident Survey, the Jefferson Scale of Empathy-Health Professionals (JSE-HP) (Hemmerdinger et al., 2007), and a questionnaire on the PI of nursing students. We obtained permission from the authors of all the published scales *via* email to use the scales for this study.

#### Sociodemographic characteristics

The following information about patients' sociodemographic and clinical characteristics was collected using a self-made questionnaire: age, gender, education level, origin of student, the only child, personality type, relationship with parents, any dispute with the patient during the internship, positions, voluntary choice of nursing profession, and degree of interest in nursing.

#### Workplace violence incident survey

The questionnaire was adapted from the hospital's WPV questionnaire that was designed by Chen (2011). The questionnaire consists of four parts. The first part includes the frequency of different types of WPV (10 items). The second part contains the victim's description of the time of the most profound WPV they had suffered during the last 6 months before the survey (including the characteristics, attitude, coping style, impact, and measures of the violence, a total of 19 items). The third part includes the cognition and attitude of interns toward WPV and coping with it (10 items). The fourth part deals with measures taken by the hospital to address WPV (8 items). The Cronbach's α coefficient of the scale was 0.986. The KMO (Kaiser–Meyer–Olkin) value was 0.948 and the Bartlett sphericity test statistic was 109,238.644 (*P* = 0.000).

#### Jefferson scale of empathy-health professionals (JSE-HP)

The English version of the JSE-HP, developed in 2001 by Dr. Mohammadreza Hojat and his research team at Thomas Jefferson University, was used to measure the healthcare workers' empathy. This scale comprises 20 items, each assessed on a 7-point Likert-type scale including three dimensions: perspective taking, compassionate care, and standing in the patient's shoes. Higher scores indicated more empathy. We used the Chinese version of this scale, which was shown to have good reliability and validity in a previous study on nursing students. The Cronbach's alpha coefficient in the present study was 0.836 (Li and Sun, 2011).

#### The scale of professional identity about nursing students

The researchers prepared and distributed a questionnaire about “the research on PI of Nursing students,” which was adapted from “the research on PI of medical students” compiled by Zhang (2010). The statistical results presented good credibility and validity. The PI of the nursing students consisted of six dimensions, which were vocational cognition (7 items), vocational emotion (5 items), vocational comments (6 items), vocational behavior (3 items), vocational expectation (3 items), and vocational values (7 items). The questionnaire contained a total of 31 items. Using a 5-point scale, the subjects were asked to choose the answer that was consistent with themselves from “very inconsistent”, “relatively inconsistent”, “no clear opinion”, “relatively consistent”, and “very consistent” in the description of the items according to their consistent situation. Among them, 5 points were allotted for clicking “very inconsistent”, 4 points for clicking “relatively inconsistent”, 3 points for clicking “no clear opinions”, 2 points for clicking “relatively consistent”, and 1 point for clicking “very consistent”. We tested the reliability and validity of the revised scale and it showed good reliability and validity. Cronbach's alpha coefficient in the present study was 0.929. The KMO value was 0.952 and the Bartlett sphericity test statistic value was 7,556.07 (*P* = 0.000).

### Data analysis

**Figure 1:**
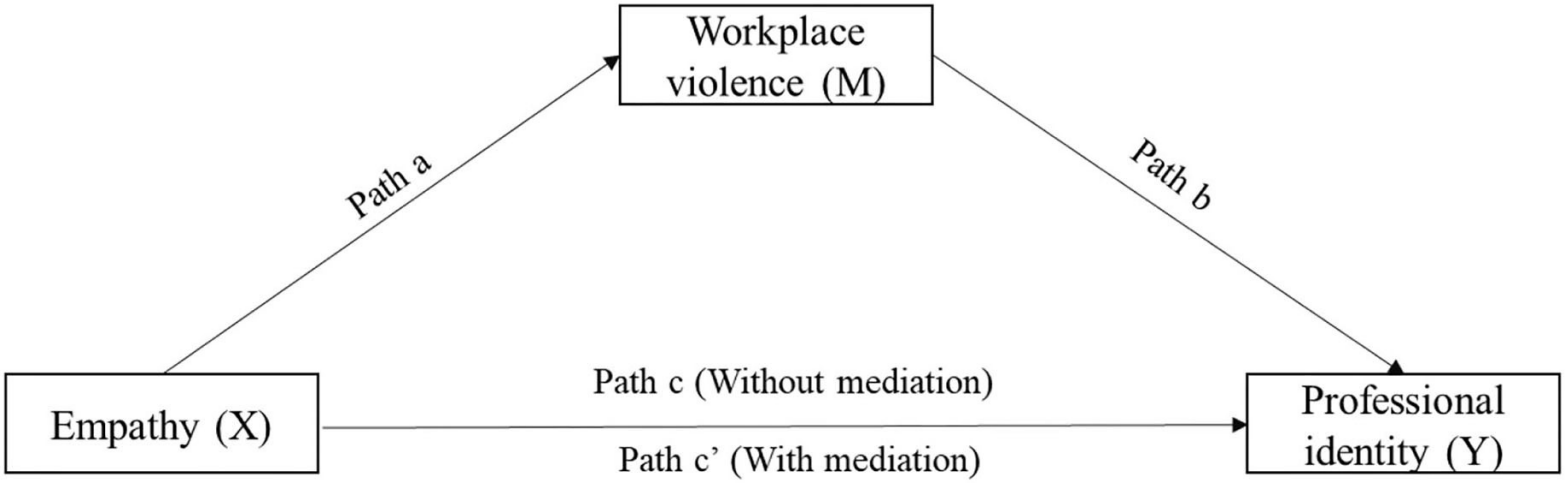
Theoretical framework of this study.

**Figure 2:**
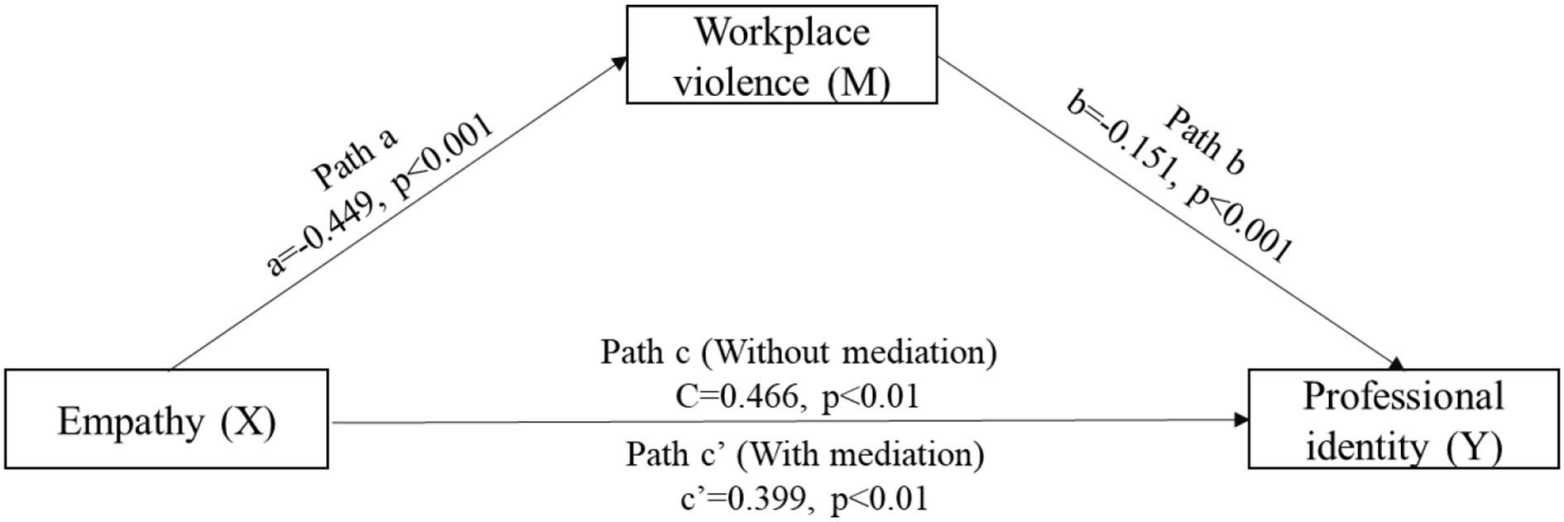
Mediating role of workplace violence in the relationship between empathy and professional identity among nursing students.

The IBM SPSS statistics software version 22.0 (IBM Corp., Armonk, NY, USA) was chosen to analyze the obtained data. All continuous variables with a normal distribution were described using means and standard deviation (means ± SD). In addition, the categorical variables were summarized by numbers or percentages. The independent-sample *t*-test or analysis of variance (ANOVA) was used to compare the scores of WPV, empathy, and PI among nursing students and sociodemographic characteristics. The correlation among WPV, empathy, and PI was determined by the Pearson correlation analysis. Additionally, the mediation analytical framework described helped to guide the analysis plan (Baron and Kenny, 1986). The capital letters X, M, and Y were chosen to represent empathy, WPV, and PI, respectively. Variable M was considered a mediator if: (1) X significantly predicted Y directly (Path c in Figure 1), (2) X significantly predicted M (Path a in Figure 1), or (3) M significantly predicted Y after controlling for X (Path b in Figure 1). Path c' meant the direct effect of X on Y after controlling for M (Path c' in Figure 1). If the regression correlation coefficient of path c' was not significant, then this mediating effect of M was complete mediation. If the regression correlation coefficient of path c' was significant, then this mediating effect of M was partial mediation. The mediation effect value was calculated as *a* ^*^ *b*, and the ratio of the mediating effect to the total effect was *a* ^*^ *b*/*c*.

## Results

### Current situation of empathy, workplace violence, and professional identity in nursing students

In total, 410 nursing students were surveyed in this study, and 405 surveys were completed, yielding a response rate of 98.78%. Demographic characteristics, empathy value, WPV, and PI scores of the studied participants are presented in Table 1. Voluntary choice of nursing profession had significantly different scores of empathy, WPV, and PI. Location of their home difference in students could lead to varying empathy levels and WPV in a significant situation. Dispute with the patient had significantly different outcomes of WPV and PI. Education levels and a strong influence on professional understanding also have significantly different effects on WPV. In addition, a good relationship with parents, interest in nursing profession, and academic achievement level have a significant influence on WPV. In addition, ninety (i.e., 22.22% of nursing students) nursing students had experienced WPV. The average scores for empathy and PI were 83.17 ± 20.71 and 103.69 ± 17.79, respectively.

**Table 1:** Scores on workplace violence, empathy, and professional identity of nursing students of different characteristics.

**Factors**	**Items**	**Workplace violence**	**Empathy**	**Professional identity**
Gender	Male	8 (23)	4.296± 1.536	3.454 ± 0.544
	Female	82 (382)	4.150 ± 1.000	3.338 ± 0.576
*F*/*X*^2^		2.226	0.449	0.942
*P*		0.136	0.658	0.347
Whether the only child	Yes	16 (58)	4.029 ± 0.900	3.297 ± 0.573
	No	74 (347)	4.180 ± 1.056	3.353 ± 0.574
*F*/*X*^2^		1.127	−1.025	−0.686
*P*		0.288	0.306	0.493
Origin of student	Rural areas	57 (302)	4.224 ± 1.030	3.351 ± 0.571
	Urban areas	33 (103)	3.967 ± 1.034	3.326 ± 0.584
*F*/*X*^2^		7.701	2.187	0.379
*P*		0.006	0.029	0.705
Conflict with patients during the internship	Yes	37 (77)	3.981 ± 1.091	3.155 ± 0.580
	No	53 (328)	4.200 ± 1.019	3.390 ± 0.564
*F*/*X*^2^		36.700	−1.677	−3.272
*P*		0.000	0.094	0.001
Internship position	Student	72 (340)	4.200 ± 0.996	3.357 ± 0.548
	Team leader	11 (37)	3.930 ± 1.037	3.303 ± 0.663
	Group leader	7 (28)	3.950 ± 1.422	3.257 ± 0.744
*F*/*X*^2^		1.547	1.754	0.501
*P*		0.461	0.174	0.606
Whether they chose nursing profession voluntarily	Yes	59 (299)	4.222 ± 1.056	3.446 ± 0.556
	No	31 (106)	3.978 ± 0.956	3.059 ± 0.528
*F*/*X*^2^		4.097	0.290	0.448
*P*		0.043	0.037	0.000
Relationship with parents	Harmoniousness	63 (278)	4.225 ± 1.094	3.393 ± 0.579
	General harmoniousness	19 (90)	3.947 ± 0.839	3.286 ± 0.510
	Ordinary	8 (34)	4.203 ± 0.992	3.028 ± 0.533
	Less harmoniousness	0 (2)	3.500 ± 0.707	4.436 ± 0.753
	Disagreeable	0 (1)	4.400	3.839
*F*/*X*^2^		0.986	1.468	5.565
*P*		0.912	0.211	0.000
The degree of interest in nursing profession	Full of interest	12 (52)	4.427 ± 1.454	3.746 ± 0.758
	Interest	37 (190)	4.145 ± 0.928	3.505 ± 0.452
	Ordinary	35 (152)	4.109 ± 0.981	3.057 ± 0.429
	Disinclination	3 (6)	4.200 ± 0.596	2.973 ± 0.986
	Dislike	3 (5)	3.340 ± 1.403	2.297 ± 0.305
*F*/*X*^2^		7.716	1.766	31.788
*P*		0.103	0.135	0.000
Education Level	Technical secondary school students	2 (6)	3.783 ± 1.097	3.344 ± 0.744
	Junior college students	70 (344)	4.186 ± 1.068	3.372 ± 0.582
	Undergraduate students	11 (41)	4.105 ± 0.625	3.187 ± 0.496
	Graduate students	7 (14)	3.796 ± 1.162	3.150 ± 0.422
*F*/*X*^2^		7.881	0.950	1.831
*P*		0.049	0.417	0.141
Whether to learn nurse-patient communication course at school	Yes	81 (378)	4.148 ± 1.028	3.347 ± 0.575
	No	9 (27)	4.298 ± 1.144	3.317 ± 0.563
*F*/*X*^2^		2.066	−0.726	0.265
*P*		0.151	0.468	0.791
Academic performance level	Excellence	16 (73)	4.162 ± 1.189	3.466 ± 0.629
	Good	43 (229)	4.212 ± 0.961	3.376 ± 0.546
	Medium	28 (94)	4.088 ± 0.965	3.219 ± 0.533
	Pass	3 (9)	3.500 ± 1.908	2.882 ± 0.831
*F*/*X*^2^		5.332	1.566	4.916
*P*		0.149	0.197	0.002
Factors influence your understanding of major during the internship	Teaching teacher	59 (274)	4.167 ± 1.067	3.360 ± 0.575
	Grades of internship hospital	4 (7)	4.114 ± 1.896	3.539 ± 0.529
	Internship department	10 (27)	4.074 ± 1.362	3.252 ± 0.763
	Practical nursing work	17 (97)	4.161 ± 0.740	3.313 ± 0.513
*F*/*X*^2^		9.681	0.069	0.665
*P*		0.021	0.976	0.574
Grades of Internship hospital	Level III A hospital	87 (387)	4.164 ± 1.038	3.357 ± 0.572
	Level III B hospital	0 (4)	4.150 ± 0.673	3.186 ± 0.590
	Level II A hospital	2 (8)	3.763 ± 0.814	3.048 ± 0.612
	Others	1 (6)	4.350 ± 1.386	3.043 ± 0.582
*F*/*X*^2^		1.301	0.460	1.434
*P*		0.729	0.711	0.232

### Correlations between workplace violence, empathy, and professional identity

The situation for the total WPV was negatively correlated with the score for the empathy-health scale at a significant level (*r* = −0.449, *p* < 0.001). The situation for the total WPV was negatively correlated with the score for the PI scale at a significant level (*r* = −0.330, *p* < 0.001). In addition, the empathy-health level was positively correlated with the score for the PI (*r* = 0.466, *P* < 0.001).

### Analysis of the mediating role of workplace violence between empathy and professional identity

Figure 2 indicates the mediating role of WPV in the relationship between empathy and PI. The results showed that after controlling for sociodemographic variables, a significant total effect of empathy on PI was identified (Path c: *c* = 0.446, *t* = 10.585, *p* < 0.001). In path a, empathy had a negative impact on WPV (Path a: *a* = −0.449, *t* = −10.080, *p* < 0.001). In path b, WPV had a negative impact on PI (Path b: *b* = −0.151, *t* = −3.098, *p* < 0.001). In addition, in path c, empathy had a positive impact on PI (Path c: *c* = 0.339, *t* = 8.170, *p* < 0.001).

The mediation effect value was calculated as −0.449^*^(−0.151), that is, 0.068, and the ratio of the mediating effect to the total effect was 14.59% (0.068/0.466 = 0.1459). A summary of the mediating effects of WPV between empathy and PI is shown in Table 2.

**Table 2:** Summary of the mediating effects of workplace violence between empathy and professional identity.

**Effect**	**Independent variables**	**Dependent variables**	***B***	***T***	***P***	***R* ^2^**	***F***
Total effect(c)	X	Y	0.466	10.585	0.000	0.218	112.050^***^
Indirect effect(a)	X	M	−0.449	−10.080	0.000	0.201	101.605^***^
Indirect effect(b)	M	Y	−0.151	−3.098	0.002	0.236	62.017^***^
Direct effect(c')	X	Y	0.399	8.170	0.000		

*** *P* < 0.001.

The regression correlation coefficients of Path a, Path b, Path c, and Path c' were all significant. Therefore, WPV had a partial mediating effect on the relationship between empathy and PI. Empathy predicted nursing students' PI partially through WPV.

## Discussion

Professional identity is theoretically a key factor in providing high-quality care to improve patient outcomes and is thought to mediate the negative effects of a high-stress workplace and improve clinical performance and job retention (Sun et al., 2016). In this study, the level of PI about nursing students is not satisfactory. This result was consistent with the findings from other studies worldwide (Guo et al., 2018; Chen et al., 2020). That could be a major problem for nursing student's employment options upon graduation. In addition, the poor level of PI aggravates the shortage of nursing staff in hospitals, increase the difficulty of nursing management, and ultimately lead to patients being unable to get good care and assistance.

Professional identity is the professional self or self-concept of nursing that represents how nurses or nursing students perceive the nursing profession to be. In our study, we found that the PI was influenced by the conflict with patients during the internship, whether they chose nursing profession voluntarily, the degree of interest in nursing profession, academic performance at school, and the degree of close relationship with parents. Another study found that demographic factors such as family residence and presence of relatives in medical service were positively related to PI (Chen et al., 2020). However, the level of PI has no significant difference between students from different family residences in our survey. Nursing college and internship are critical periods when the PI is formed (Chen et al., 2020). Effort taken to improve nursing students' PI is an ongoing process and it is necessary to arouse the common attention of educators, clinical managers, and society.

Empathy is an underlying foundation for nursing. Having nursing knowledge alone is just not sufficient for nurses to care for patients and their families (Li et al., 2019). Patients and their families value not only nurses' expertise but also their empathetic behavior (Wei et al., 2018). So, the total score for the value of empathy was 83.17 ± 20.71 in our study. This result indicates that the level of empathy of nursing students is low. It can be seen that the level of nursing students' ability of empathy is not satisfactory. While the ability of empathy has a significant positive effect on PI, which is consistent with the findings of other researchers (Percy and Richardson, 2018; Messineo et al., 2021). Thus, we need to pay more attention to this aspect. Moreover, empathy can effectively improve the relationship between the patients and the doctors and enhance patient satisfaction (Petrucci et al., 2016).

In the previous study, the overall mean empathy scores for undergraduate nursing students were lower than those reported in studies conducted in Western countries (Candilis, 2002; Bas-Sarmiento et al., 2017). More and more studies are showing that it is imperative to pay attention to the cultivation of nurses' empathy value (Díaz Valentín et al., 2019; Di Lorenzo et al., 2019). Another study examined the empathy levels of students who were enrolled in different health disciplines from two large Australian universities. The results of the findings of that study revealed that paramedic students had statistically lower empathy results than all other health professions, except nursing students (Brett et al., 2014). Besides, female students reported a higher mean score on the JSE (Piumatti et al., 2019; Messineo et al., 2021). McKenna et al. also looked at empathy levels in nursing students and found that there were no significant differences in empathy relating to age, sex, or year of study (McKenna et al., 2011; Brett et al., 2014), which is quite similar to the results found in our research. While a recent study suggests that empathy levels decline as a student progresses through a program (Paula et al., 2011). It would be worth exploring the innumerable factors that contribute to these results, such as experience gained during student placements and mentoring during study.

In the present study, students who choose to engage themselves in the nursing profession tended to have higher empathy levels and PI. We speculate that students who choose nursing majors voluntarily have a better understanding of professional connotation, which is of great significance to the formation of PI. Therefore, nursing students who are not choosing nursing majors voluntarily should be targeted for empathy and PI improvement. We also found that those who were born in the countryside have higher empathy levels, one possible explanation is that children born in rural areas have higher levels of resilience and dare to face life's challenges. Moreover, children who grow up in the countryside have more freedom to play with their peers and have a more harmonious neighborhood.

Furthermore, empathy was found to be positively correlated with PI for nursing students in the present study. This was consistent with the study about graduate students (Dobrowolska et al., 2014). It has been explicitly demonstrated that empathy strengthens the relationship between patients and health professionals and also improves their satisfaction, which in turn helps to promote the best clinical outcomes (Petrucci et al., 2016). In contrast, inadequate levels of empathy shown by the nursing students could result in an unsatisfactory result in both patients and health professionals (Doyle et al., 2014; Ferri et al., 2015).

The WPV phenomenon is prevalent in various nursing clinical settings (Edward et al., 2016). About 22.22% of the nursing students experience WPV as reflected in our study. WPV was found to be negatively correlated with PI for nursing students. In addition, WPV was also confirmed to be an independent predictor of PI in the present study. This result was consistent with that of the previous study (Luthans and Youssef, 2004). One of the proper explanations put forth is: when experiencing WPV physically or mentally, nursing students suffer from poor productivity, a lower quality of work, and a decline in individual sense of accomplishment. Students with an experience of WPV are more likely to display a lower PI. In other words, nursing students who have not experienced WPV were more likely to choose a career as a nurse. Possible reason might be attributed to the fact that nursing students who had already experienced WPV think of nursing to be a dangerous profession. A similar study about the doctor arrived at the same opinion that WPV was negatively related to PI (Qiu et al., 2019). Moreover, we found that birthplace, conflicts with patients, voluntary selection of nursing profession, different levels of education, and a strong impact on the professional understanding during their internship were all closely related to WPV. Interestingly, a study about the nurses who were the only children in their families tended to have higher odds of experiencing physical and non-physical violence (Zhang et al., 2017). However, we found no difference in this aspect.

Workplace violence was found to be a partial mediator in the relationship between the levels of empathy and PI in the present study and the mediating effect value was 14.59%. Consequently, WPV exerted a significant effect on partially mediating the association between the levels of empathy and PI, despite a high level of empathy including three factors in the process of choosing nursing being extremely important for nursing students to have a better PI, while WPV also played a critical mediating role in curbing the PI. A possible explanation for this interaction is that optimal levels of empathy could be basic essentials for nursing students to have a better PI, but not experience WPV, even if they convince themselves to believing that their choice to be a nursing student is the right step in the right direction. Therefore, WPV is a vital mediating predictor of PI.

Based on the results of this study, we put forward some valuable suggestions which are of immense benefit, from two aspects, to improve the nursing students' PI. First, effective interventions to improve nursing students' levels of empathy should be designed and implemented. When more and more researchers evinced a strong interest in this direction and pay adequate attention to improving nursing students' levels of empathy, they argue, can empathy be improved with certain interventions (Bas-Sarmiento et al., 2017; Ding et al., 2020; Peng et al., 2020). Teachers should pay more attention to cultivating students' levels of empathy. Nursing educators should focus more on the formation of the students' PI and caring as a contributing factor to it. Second, we found that WPV also had an important effect on PI. Maybe, the occurrence of WPV can be effectively reduced from the following aspects. For nursing students, it is imperative that they need to learn to identify the factors associated with WPV and report them on time. For nursing educators and clinical managers, it is imperative that they inform students about the WPV preventive measures and provide them with a safe working environment. The last, health organizations must act to examine how cases of WPV against students are handled (Warshawski, 2021).

## Conclusion

Our study demonstrates that WPV has a partially significant mediating effect on the relationship between the levels of empathy and PI. Nursing educators should pay attention to the cultivation of students' levels of empathy. Considering the prevalence of low level of PI among nursing students, targeted interventions to protect them from WPV could increase the chances of choosing a nursing career. WPV is common among nursing students, which has a huge impact on PI. On the one hand, practice hospitals should strengthen the protection of interns; on the other hand, schools and practice hospitals should strengthen the recognition and training of interns, especially those with low empathy, in dealing with WPV.

This study, however, has some limitations which need to be illustrated. First, although questionnaires about WPV and PI have good reliability and validity, they have not been tested by using large samples. The results of this study should be confirmed by a longitudinal study. Second, we selected nursing students only from five universities in Hunan Province. Therefore, the sample may not be completely representative of all Chinese nursing students. Lastly, we will extend the investigating areas in the future research.

## Implications for nursing management

Although the hospitals are facing the shortage of nurses, more and more nursing students are escaping from nursing work in China. Workplace violence, professional identity, and empathy are the important factors that influence the nursing students' decision in whether to choose nursing as their career or not. The low professional identity and workplace violence are the important reasons for nursing students not choosing nursing as their careers. While empathy is positively correlated with professional identity and can improve the professional identity of the nursing students, it also encourages nursing students to continue with the nursing job. Therefore, the mediating effect of workplace violence on the relationship between empathy and professional identity among nursing students is studied. This study can guide nursing managers to take targeted measures at improving the professional identity of nursing students to enable them to wholeheartedly engage in nursing work, thereby contributing to patient care and welfare.

## Data availability statement

The raw data supporting the conclusions of this article will be made available by the authors, without undue reservation.

## Ethics statement

The studies involving human participants were reviewed and approved by The Second Xiangya Hospital's Ethics Committee. The patients/participants provided their written informed consent to participate in this study.

## Author contributions

LZ and LW: conceptualization, methodology, and data curation. CF, HL, QC, and LC: formal analysis. LW: writing—original draft preparation and writing—review and editing. All authors have read and agreed to the published version of the manuscript.
